# Extracting Crystal Chemistry from Amorphous Carbon Structures

**DOI:** 10.1002/cphc.201700151

**Published:** 2017-03-08

**Authors:** Volker L. Deringer, Gábor Csányi, Davide M. Proserpio

**Affiliations:** ^1^ Engineering LaboratoryUniversity of CambridgeTrumpington StreetCambridgeCB2 1PZUnited Kingdom; ^2^ Department of ChemistryUniversity of CambridgeLensfield RoadCambridgeCB2 1EWUnited Kingdom; ^3^ Università degli Studi di MilanoDipartimento di ChimicaMilanoItaly; ^4^ Samara Center for Theoretical Materials Science (SCTMS)Samara UniversitySamaraRussia

**Keywords:** ab initio calculations, carbon allotropes, high-throughput screening, machine learning, solid-state structures

## Abstract

Carbon allotropes have been explored intensively by ab initio crystal structure prediction, but such methods are limited by the large computational cost of the underlying density functional theory (DFT). Here we show that a novel class of machine‐learning‐based interatomic potentials can be used for random structure searching and readily predicts several hitherto unknown carbon allotropes. Remarkably, our model draws structural information from liquid and amorphous carbon exclusively, and so does not have any prior knowledge of crystalline phases: it therefore demonstrates true transferability, which is a crucial prerequisite for applications in chemistry. The method is orders of magnitude faster than DFT and can, in principle, be coupled with any algorithm for structure prediction. Machine‐learning models therefore seem promising to enable large‐scale structure searches in the future.

Exploring structural space—of allotropes, polymorphs, materials—is today not only done experimentally but also by computational techniques and in an increasingly automated fashion.^1^ Indeed, with advanced algorithms and high‐performance computing centres available, ab initio crystal structure prediction methods revealed novel and intriguing structures of elements and compounds, including stoichiometric compositions and coordination modes that would not have been expected from textbook knowledge. Many of these predictions were subsequently validated by experiments.^2^

Among the elements, carbon is one of the structurally most diverse, and naturally has long been the target of crystal‐chemical considerations^3^ and later of structure‐searching algorithms. New carbon allotropes have been predicted using practically every computational method available, including ab initio random structure searching (AIRSS),^4^ genetic algorithms,^5^ particle swarm optimization,^6^ metadynamics,^7^ and minima hopping.^8^ A recent, critical survey of the field is in Ref. 9.

Despite their predictive power, ab initio structure searches are inherently limited by the underlying computational workhorse, most commonly density‐functional theory (DFT), which becomes prohibitively expensive for larger system sizes. To overcome the latter, more general problem, a novel class of interatomic potentials based on machine learning (ML) is currently emerging in the solid‐state theory communities.^10^ Such ML potentials are trained on DFT or other quantum‐mechanical data, and in doing so provide a high‐dimensional fit of the potential‐energy surface. These potentials enable simulations that can come close to DFT accuracy, but are faster by many orders of magnitude; they are still much slower than established empirical force fields, but the trade‐off is often worthwhile. For example, an ML‐based neural‐network potential enabled realistic, atomic‐scale insight into the graphite‐diamond transition, learning from DFT computations on structural snapshots taken from graphite, diamond, and intermediates.^11^ It was recently suggested that ML potentials might be beneficial for structure searches.^12^

Herein, we show that structural information from liquid and amorphous forms of carbon can be harnessed, via machine learning, to guide searches for crystalline phases (Figure 1). This serves as proof‐of‐concept that ML models, if properly trained, can indeed be used for applications in solid‐state chemistry, including the exploration of (previously unknown) structural space.

**Figure 1:**
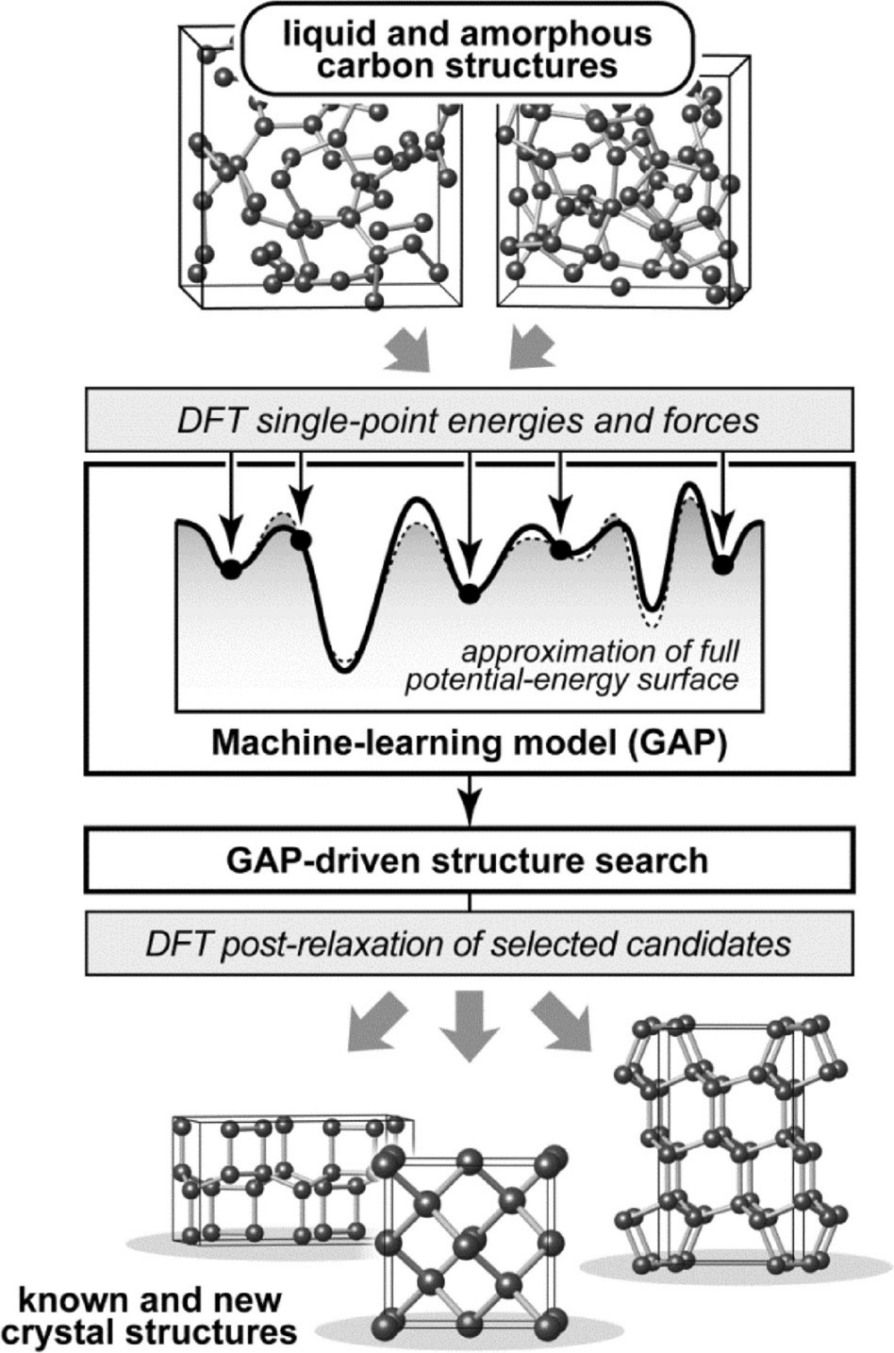
Flowchart of the strategy employed in the present work. Starting from liquid and amorphous carbon structures, we generate a Gaussian approximation potential (GAP) similar to that in Ref. 13 but here excluding any crystalline training data on purpose. We then use this for random structure searching,1d and subsequently re‐relax suitable candidate structures with DFT (see text).

To validate our approach, we performed a numerical experiment, starting with a set of of fully DFT‐driven structure searches1d using 1,000 randomized unit cells that each contained eight carbon atoms. DFT relaxation of these cells readily identified diamond and graphite, and also several less stable structures with mixed coordination numbers, all as expected (Figure 2 a). We then performed Gaussian approximation potential (GAP)‐driven searches, starting from the same initial configurations and probing how close the results would come to DFT. Initially, this led to a set of structures slightly higher in energies than the reference data, but this can be easily remedied by a subsequent DFT relaxation (Figure 2 a, left). Likewise, the distribution of optimized volumes is similar for the DFT‐ and GAP‐based procedure (right). This justifies our strategy: we perform large numbers of GAP‐driven searches, and then re‐relax only the most promising candidates using DFT.

**Figure 2:**
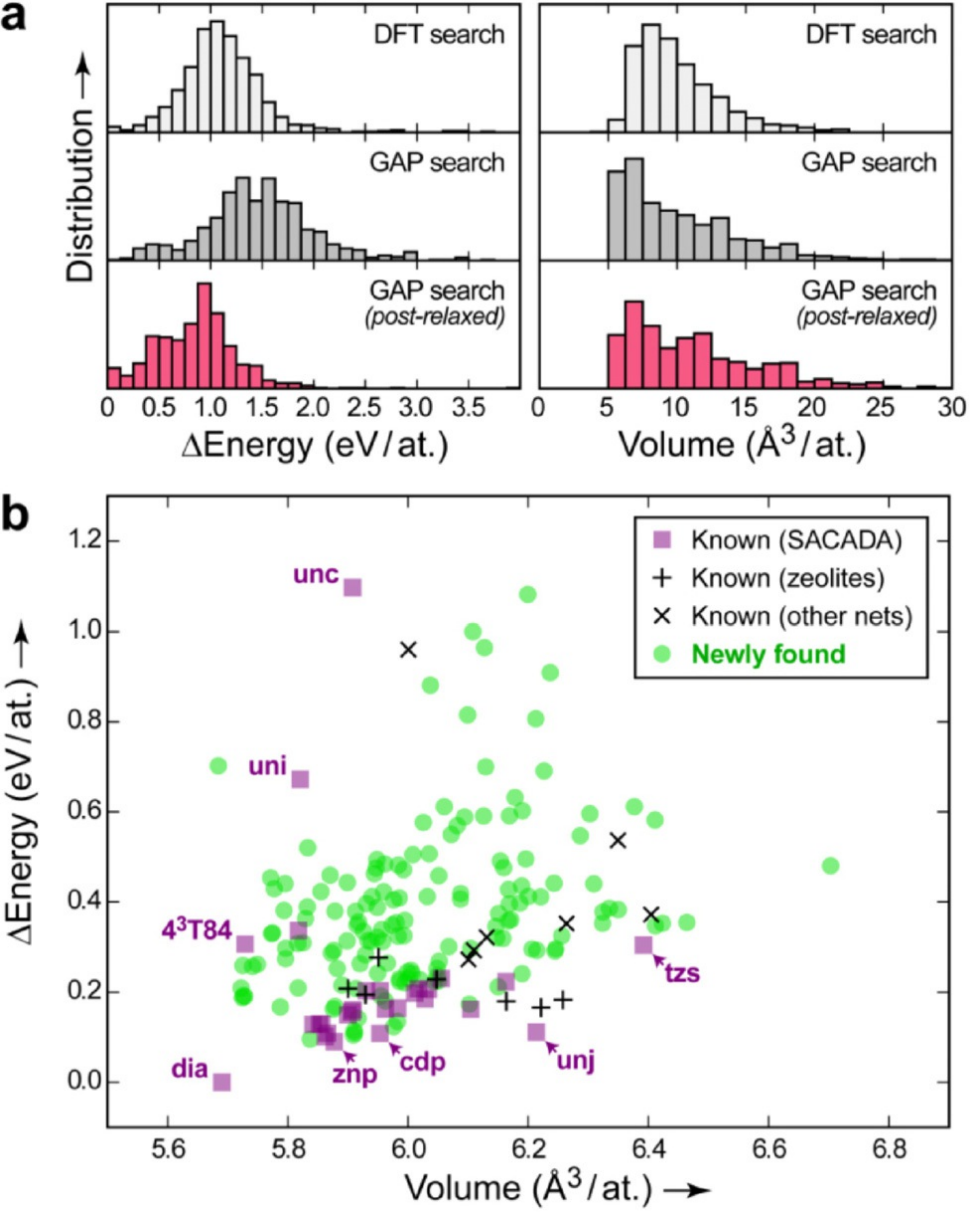
a) Tests of energy and volume distributions from a trial set of DFT‐ and GAP‐driven structure searches. To enable comparison, all energies given have been re‐computed using DFT. b) Energy–volume plot for the results of our main, much larger structure search, including known (purple, black) and new (green) carbon allotropes. Topology symbols^14^ such as „**dia**“ are given for a number of representative known structures. Our search also found lonsdaleite (**lon**) and a mixed **dia**/**lon** stacking sequence that are omitted for clarity; detailed results are given in the Supporting Information.

The main result of this work is summarized in Figure 2 b. We have here performed a large‐scale search for allotropes with fourfold coordination exclusively, but we stress that our GAP model can describe mixed coordination environments just as well.^13^ Our search yielded 197 distinct carbon networks which were classified according to their topology;^14^ they were checked against the Samara Carbon Allotrope Database (SACADA; Ref. 9) and furthermore against other topological nets as collected in ToposPro TTD;^14^ some of these were seen in zeolites or metal‐organic frameworks but not in carbon allotropes. These structures are considered known and here referred to as such.

In addition, our search returned 150 possible allotropes that are neither known to SACADA nor from other topology databases; of these, 52 are no more than 0.3 eV per atom (≈30 kJ mol^−1^) above diamond in their DFT‐computed energy. Many of these structures are best understood by dissecting them into characteristic, topological building blocks.^15^, ^16^ For example, carbon atoms in diamond form six‐membered rings exclusively, and four of these combine into an adamantane‐like cage, such that the tiling symbol for **dia** is written as [6^4^] (details may be found in Ref. 17). It was previously pointed out how other structural motifs can be combined with **dia** (or lonsdaleite, **lon**) cages to form more complex allotropes.8b, 15 Figure 3 a illustrates this using an example: combining **dia**, **lon**, and the characteristic five‐ and seven‐membered ring fragments of **cbn** (M‐carbon; Ref. [5a‐b]) leads to a new structure, **G95**, that is found by our search. (We label all new structures with a G for GAP, and the number is simply a running index).

**Figure 3:**
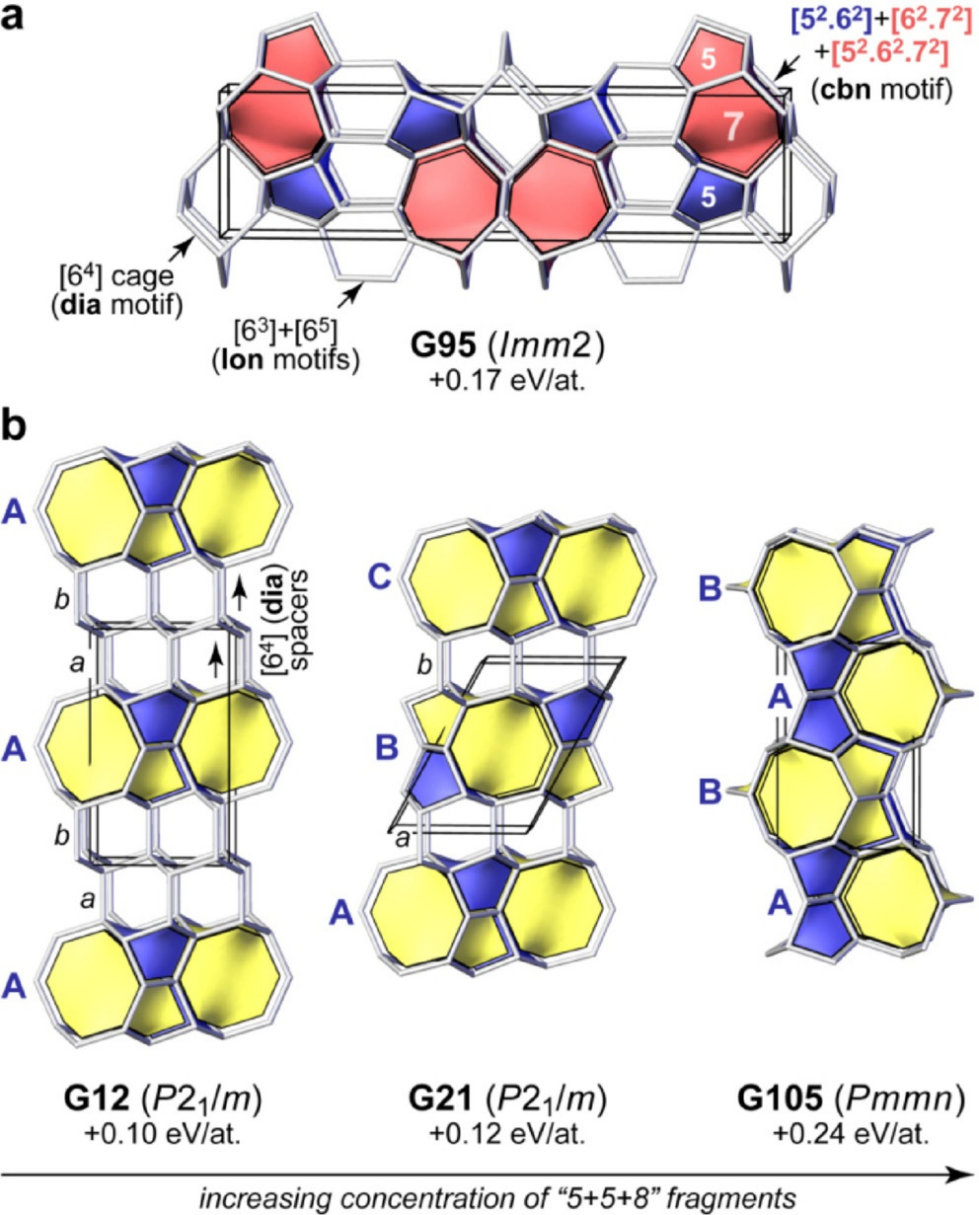
a) **G95**, a predicted carbon allotrope that combines structural motifs from diamond (**dia**), lonsdaleite (**lon**), and „M‐carbon“ (**cbn**), as revealed by topology analysis. b) Predicted carbon allotropes with „5+5+8“ building blocks. Cages that involve an eight‐membered ring, typically [5^2^.6^2^.8^2^], are highlighted in yellow; [5^2^.6^2^] cages are blue. Interestingly, the apparent end‐member of this series, **G105**, is different in cage topology from the others, and filling space exclusively with [5^2^.6^2^.8^2^] and [5^2^.6^2^] cages would yield the **bik** network instead (Supporting Information). All energies are given relative to diamond.

We also find several new 5+5+8 allotropes8b that contain, as the name suggests, sets of five‐and eight‐membered ring fragments (Figure 3 b). In **G12**, layers of such motifs (blue/yellow) are interwoven with two consecutive layers of **dia** spacers (empty). The stacking sequence of the building units can be written as A*ab*A*ab*, where capital letters denote the stacking of the defining ring structures, and lowercase italics refer to the spacers. Reducing the concentration of the latter, we have **G21** (and **G6**, which is similar but with a less favourable stacking sequence). We also find a corresponding structure without any diamond‐like cages, **G105**, which is higher in energy as there is no dilution by **dia** spacers. In general, no straightforward correlation exists between the simplicity of the structures and their stability (Supporting Information, Figure S8).

As pointed out by Botti et al.,8b one may freely add more and more **dia** (or **lon**) like spacers to such structures, and therefore create infinite numbers of topologically unique networks. Where is the limit? Figure 3 b suggests a possible answer: we believe that truly distinct carbon allotropes should be restricted to cases with clear and simple stacking sequences both for the mixed‐ring units and the spacers. For the same reason, we have excluded a combination of only **dia** and **lon** cages from the plot in Figure 2 b, as an infinite number of similar polytypes can be trivially defined.

The critical reader will now ask whether one needs high‐throughput computations to devise such simple stacking sequences. Indeed, the true strength of GAP‐driven structure searching is that due to its speed (our search comprised over 290,000 runs), it is likely to unveil more complex cases that depart from previously established structural principles but are still energetically viable.

In the latter category fall carbon allotropes with what we call pseudo‐tiling patterns (Figure 4). Drawing 2D projections of such structures gives the impression of very small, three‐ and four‐membered rings—but in fact the relevant atoms lie atop each other along the viewing direction, and so only create the illusion of touching. Figure 4 a illustrates this for **4^3^T143**, a variant of the chiral **unj** net^18^ that has been observed in a database of hypothetical zeolites;^15^, ^19^ we note that the same topology was very recently described for Si allotropes.^16^ In **unj**, chiral tubes of fivefold rings form what looks like a honeycomb structure when viewed down the tube axis (Figure 4 b).^18^ Similar tubes exist in **4^3^T143** but there they form 2D sheets (in the *ab* plane), and these are then stacked perpendicularly along *c*. Hence, the fivefold rings seen in Figure 4 c are the actual structural motif, observed when a tube is looked upon in side view. Both structures are generated by filling space with the same topologically unique cage, [5^2^.8^2^].

**Figure 4:**
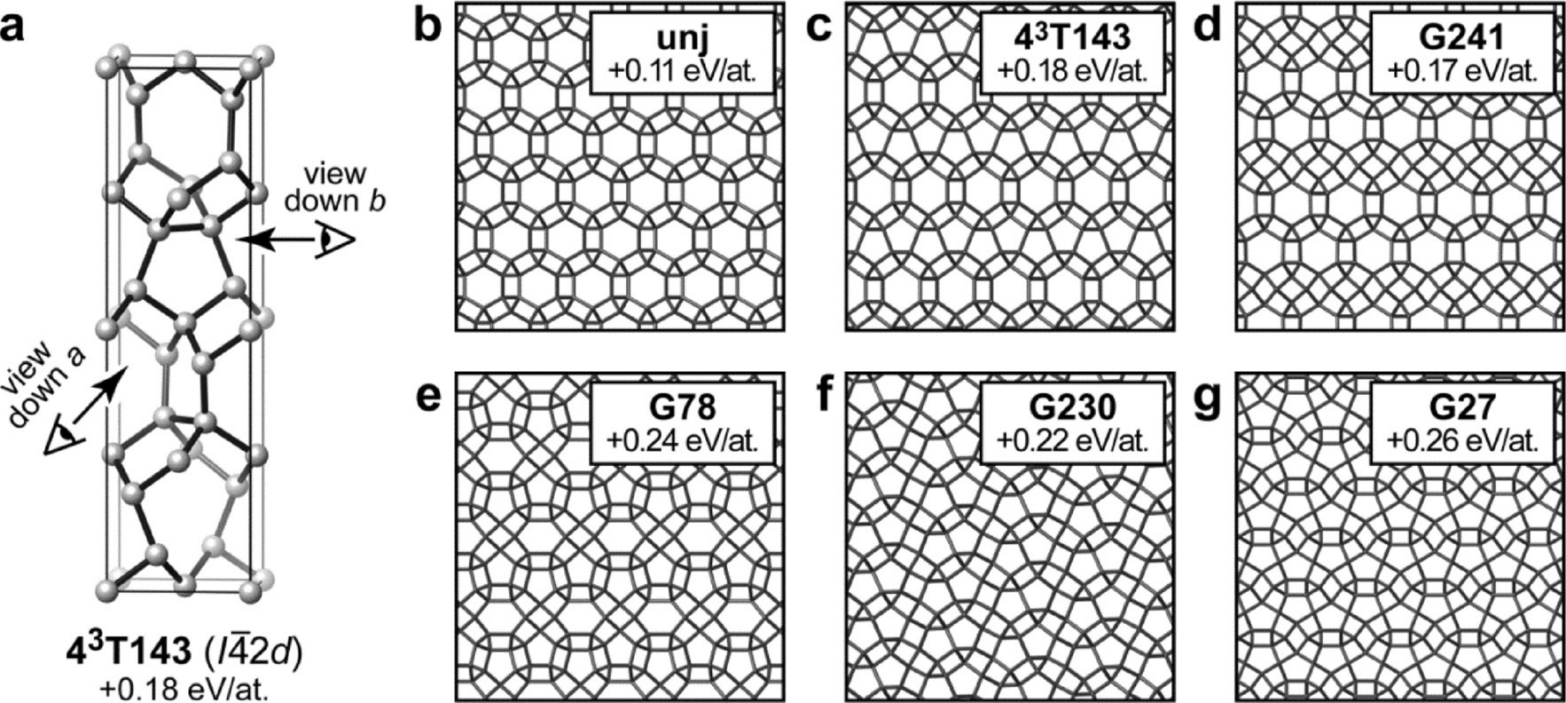
a) **4^3^T143**, a variant of the chiral **unj** framework: the structure is composed of five‐membered rings, but viewing it down the *a* or the *b* axis as indicated creates the impression of three‐, four‐, and six‐membered ones. b–g) Projection views of known and new structures found in our search.

Just like combinations of small polygons can create diverse patterns, the putative family of pseudo‐tiling allotropes is here found to span a wider range. Figure 4 d shows **G241**, a related motif in which **unj**‐like [5^2^.8^2^] cages are mixed with **dia**‐like [6^4^] ones; Figure 4 e shows **G78** which adds cages with seven‐membered rings instead; both structures are chiral in space group *C*222_1_. There are further pseudo‐tiling patterns without apparent six‐rings, as we find for **G230** and **G27** (Figure 4 f,g); both are loosely reminiscent of the *P*4_1_2_1_2 spiral structure for group‐14 allotropes predicted recently from AIRSS (4^3^T130 in SACADA).4b All these structures need not only be hypothetical constructs: recent experiments showed that complex Si allotropes of such type may indeed be formed in “microexplosions“, locally induced in a crystalline matrix by ultrashort laser pulses.^20^

We stress that AIRSS, like all ab initio methods, can only span a certain subspace: that of structures with a small number of atoms in the primitive unit cell (here, ≤16). By contrast, novel carbon allotropes have been predicted based on chemical knowledge, deriving them from zeolites^15^, ^21^ or clathrate structures;^22^ these are often highly competitive in energy, but inaccessible to DFT‐based searches. In the future, ML‐based techniques might enable the ab initio prediction of networks with hundreds of atoms in the primitive cell. And in the end, the crucial task will be not only to generate large numbers of structures out of the infinitely many possible ones (which a machine can do), but to derive new chemical insight and guidelines for experiments (which a machine cannot).

In conclusion, we have explored the structural space of carbon allotropes by combining random structure searching with an efficient machine‐learning based interatomic potential. Our GAP model readily enables predictions of crystalline phases, despite having been trained on liquid and amorphous structures alone. This represents a hard test in terms of transferability, and it opens up the road for further applications of ML models in solid‐state chemistry—where the ability to assemble and correctly describe new structures is paramount. We focused on one particular structure‐prediction method, but the ML model might just as well be coupled to others (such as genetic algorithms), or even to the nested‐sampling technique to assess temperature‐pressure phase diagrams fully from first principles.^23^ Likewise, the field of organic crystal‐structure prediction might benefit from similar techniques,^24^ albeit in that case the focus is on long‐range dispersion interactions rather than on the making and breaking of covalent bonds.^25^ Further work will extend our present findings to carbon allotropes at (very) high pressure, to networks with mixed coordination numbers, and to other materials for which similar approaches seem promising.

## Computational Methods

A GAP model10b was fitted to DFT energies and forces using the same protocols and parameters as outlined in our preceding, more technical work in Ref. 13. The input for this was a database of 3,070 liquid and amorphous carbon configurations taken from Ref. 13. Using this GAP, a total of 290,885 relaxations were performed for randomized cells containing 3–16 atoms, at ambient and elevated pressure. No symmetry operations were applied during the search itself, to allow for maximal degrees of freedom; instead, space‐group symmetry was determined a posteriori. Only structures with fourfold coordinated atoms are reported (determined using a cutoff of 1.70 Å), and candidates with three‐membered carbon rings were discarded due to the associated large strains. Post‐processing of remaining candidate structures was done by full DFT‐GGA^26^ relaxation of lattice parameters and atomic positions to zero external pressure, using CASTEP;^27^ the final structures are provided as Supporting Information in CIF format. Symmetry analyses were done as implemented in PHONOPY,^28^ PLATON,^29^ and SYSTRE;^30^ structures were visualized using GAVROG (www.gavrog.org) and VESTA.^31^

## Supporting information

As a service to our authors and readers, this journal provides supporting information supplied by the authors. Such materials are peer reviewed and may be re‐organized for online delivery, but are not copy‐edited or typeset. Technical support issues arising from supporting information (other than missing files) should be addressed to the authors.

Supplementary

Supplementary
