# Supplementary material for: Extracting Crystal Chemistry from Amorphous Carbon Structures
Source: Chemphyschem. 2017 Mar 8;18(8):873–7. doi: 10.1002/cphc.201700151 (PMC5413819; doi:10.1002/cphc.201700151)
Supplement: Supplementary file 1 — Supplementary [file CPHC-18-873-s001.pdf]

CHEMPHYSCHEM

Supporting Information

Extracting Crystal Chemistry from Amorphous Carbon Structures

Volker L. Deringer,*[a, b] Gábor Csányi,[a] and Davide M. Proserpio*[c, d]

cphc_201700151_sm_miscellaneous_information.pdf
cphc_201700151_sm_miscellaneous_information.txt

This Supporting Information document contains:

Supplementary discussion I *(further relevant, low-energy structures)* S2

Supplementary discussion II *(quantitative analyses for the full dataset)* S5

Overview of all structures found S8

Structural data for all 197 allotropes are furthermore provided in CIF format as online Supporting Information.

Supplementary discussion (Part I: further relevant, low-energy structures)

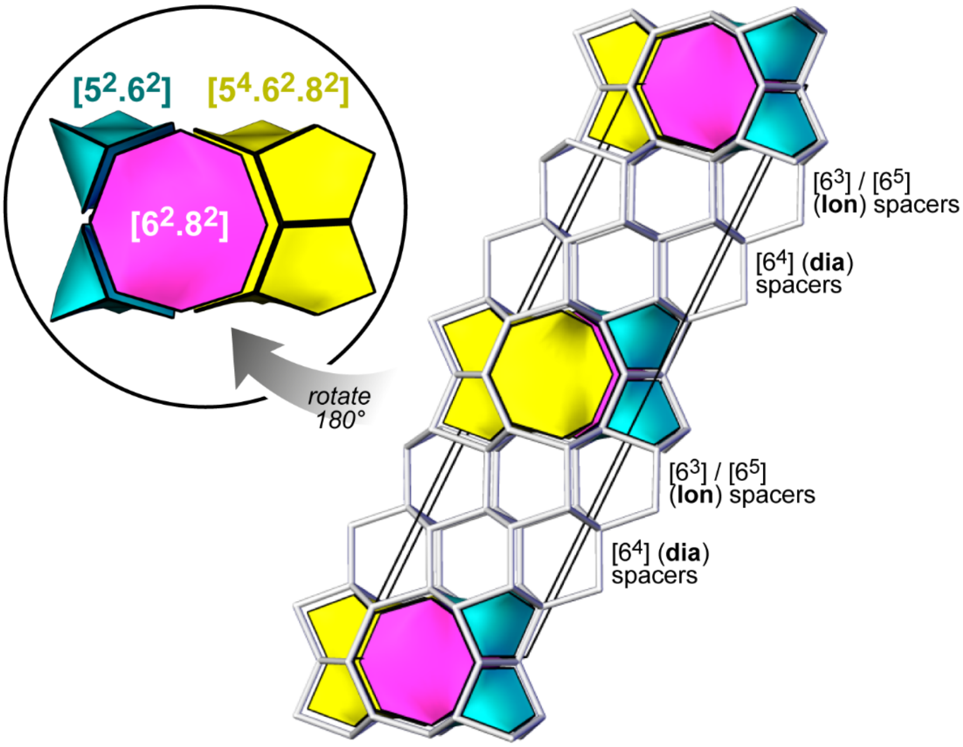

G14 (*Cm*)
+0.11 eV/at.

Figure S1: A more complex "5+5+8" structure. In the main text (Figure 3b), we discuss simple carbon allotropes containing five- and eight-membered rings. The present example, **G14**, combines many pertinent features and is therefore visualized here: it contains a combination of dia and lon spacer units, rather than one type exclusively; it also forms the "5+5+8" motif from three different cages, which are shown as close-up. The total tiling symbol for this structure is written as "$2[6^3]+2[5^2.6^2]+4[6^4]+[6^2.8^2]+2[6^5]+[5^4.6^2.8^2]$".

The complexity is also mirrored in the transitivity symbol **[(14)(21)(19)(12)]**, which means that the net contains **14** independent nodes, **21** independent edges, **19** independent five-, six-, or eight-membered rings, and **12** crystallographically distinct cages (of which there are **6** topologically distinct kinds, as reported in the tiling symbol above). Tiling and transitivity symbols for all structures are provided in Table S1 below.

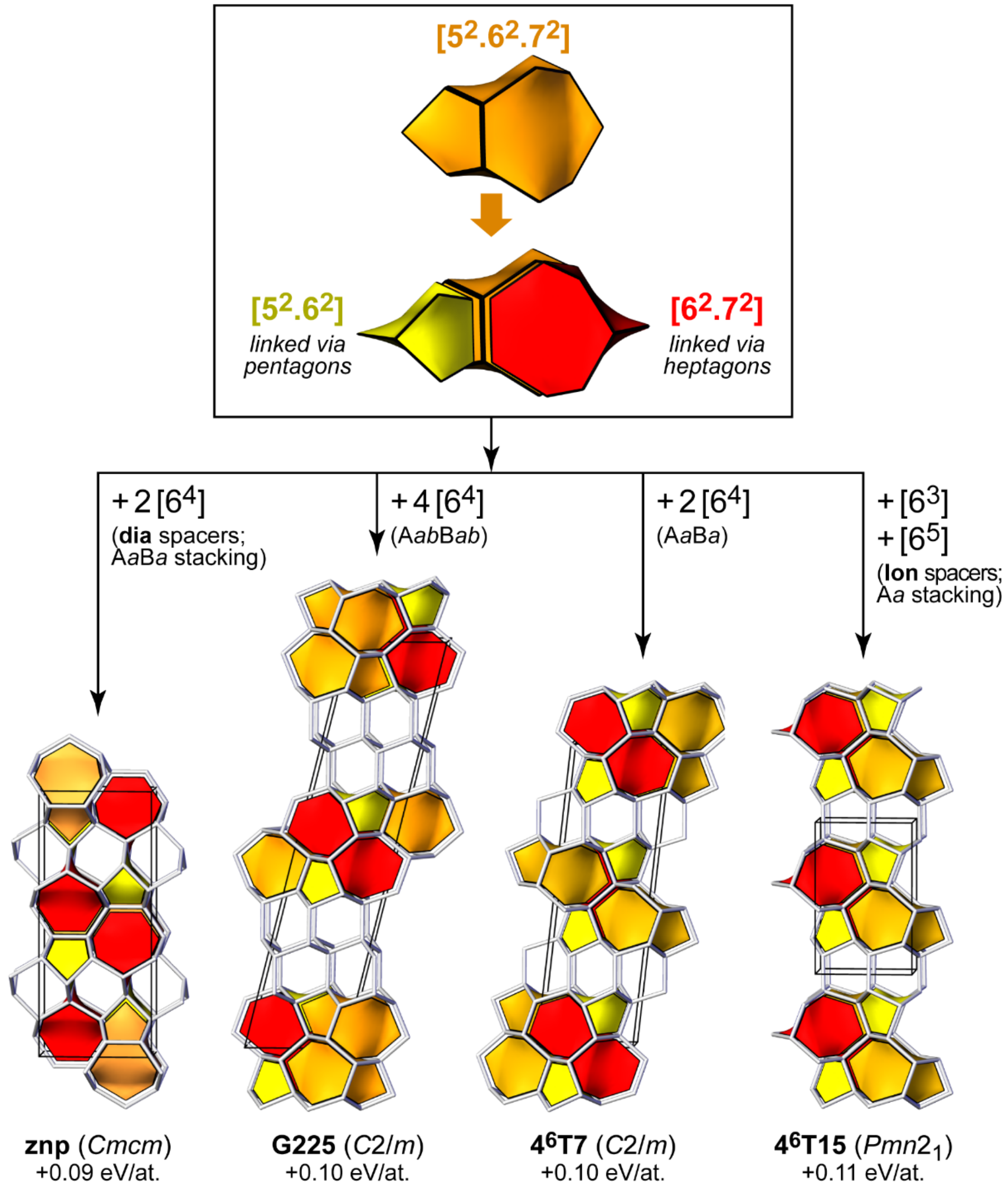

	znp	G225	4^6T7	4^6T15
transitivity:	[4664]	[8(14)(12)7]	[6(10)(10)5]	[6985]
tiling:	$[5^2.6^2]+2[6^4]+[6^2.7^2]+[5^2.6^2.7^2]$	$[5^2.6^2]+4[6^4]+[6^2.7^2]+[5^2.6^2.7^2]$	$[5^2.6^2]+2[6^4]+[6^2.7^2]+[5^2.6^2.7^2]$	$[6^3]+[5^2.6^2]+[6^2.7^2]+[6^5]+[5^2.6^2.7^2]$

Figure S2: znp and its structural relatives. The **znp** structure is the lowest-energy network (besides **dia**/**lon** and their polytypes) found in our search. The structure contains three types of cages, and they are linked as shown above (yellow, orange, and red, respectively); similar to what we discuss in the main text, these fragments can be combined with **dia** or **lon** spacers to form various related structures. The topological information for all of them is provided below: the transitivity is given in green, and the tiling symbols in blue (see also Tables S1–S3).

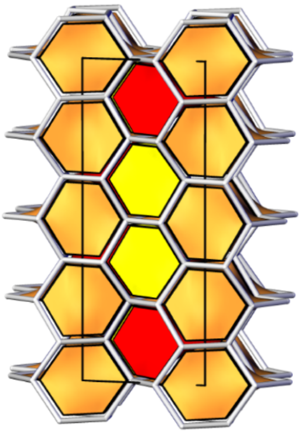

G128 (*C*2/*m*)
+0.18 eV/at.

[5(12)(10)4]

$[5^2.6^2]+[6^2.7^2]+[5^2.6^2.7^2]$

Figure S3: An un-diluted relative of znp. This structure is the lowest-energy network newly found here that does not contain any **dia** or **lon** cages. The tiling pattern is colour-coded as in Figure S2; the same tiling but with a different linkage is found in **cbn**.

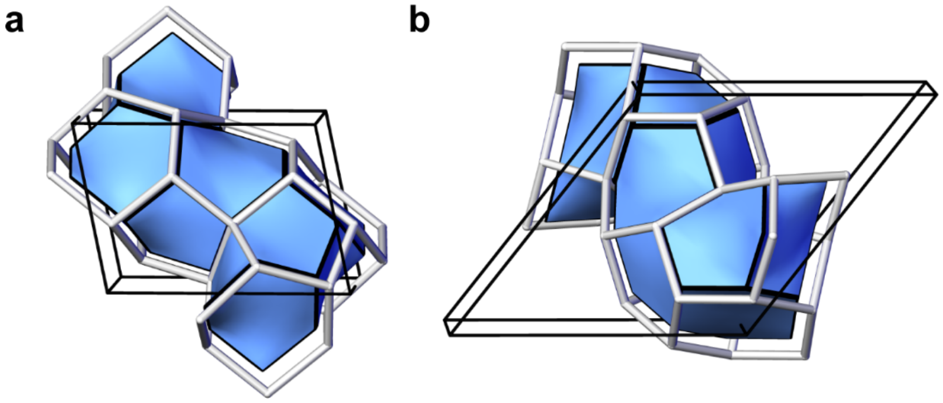

4^4T85 ($P2_1/c$)
+0.18 eV/at.

transitivity: [4951]; *tiling:* $[5^8.6^{10}]$

G230 ($P2_1/c$)
+0.22 eV/at.

transitivity: [4951]; *tiling:* $[5^8.6^{10}]$

Figure S4: Two structures from the same, complex tile. *Left:* **4^4T85**, a structure built from filling space with one type of tile, $[5^8.6^{10}]$, exclusively. *Right:* **G230**, an alternative built from the same type of tile. Only one cage is shown in each figure (the five- and six-membered rings are clearly visible), along with the boundaries of the conventional unit cell.

Supplementary discussion (Part II: quantitative analyses for the full dataset)

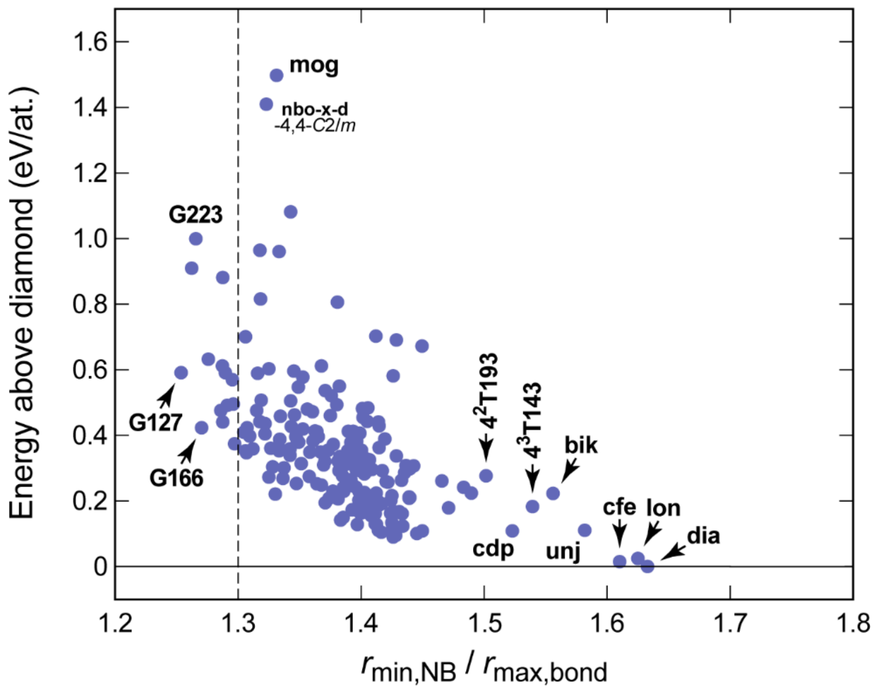

Figure S5: Ratio of shortest non-bonding to longest bonding distance: This type of analysis, as well as the deviation from ideal tetrahedral structural environments (see below) can be linked to energetic stability, as discussed in L. Öhrström, M. O'Keeffe, *Z. Kristallogr.*, **2013**, *228*, 343–346 (Ref. [3d] in the main article). Labels are given for some representative structures (*cf.* Tables S1–S3).

Below a value of ≈ 1.3 (indicated by a vertical dashed line), no low-energy structures are found. In fact, this ratio is largest overall for the diamond structure (**dia**), where the optimized covalent bond length is $r_{C–C}$ = 1.546 Å, and the next-nearest neighbour distance amounts to $r_{C\cdots C}$ = 2.525 Å, leading to a ratio of 1.63.

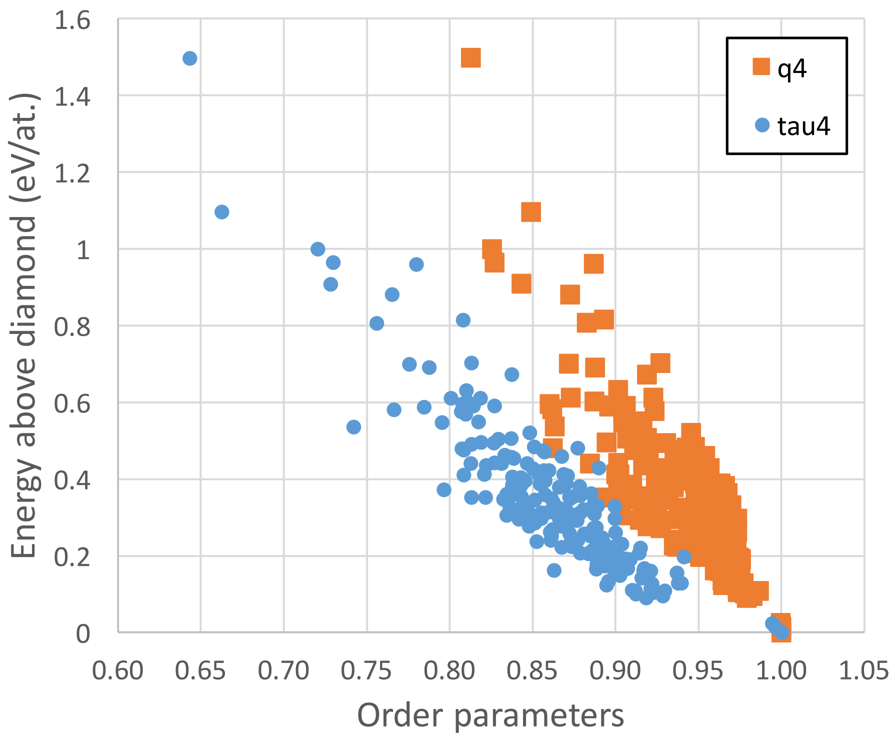

Figure S6: Structural order parameters and their relation to energetic stability. We examine two parameters, both of which are defined such that they approach 1.0 for perfect tetrahedral coordination:

- The τ_4 parameter, introduced initially for coordination compounds [L. Yang, D. R. Powell, R. P. Houser, *Dalton Trans.* **2007**, 955–964]:

$$\tau_4 = \frac{360° - (\alpha + \beta)}{141°}$$

where α and β are the two largest angles within a four-coordinate unit; if the latter is a perfect tetrahedron, then $\tau_4 = (360° - 109.5° - 109.5°)/141° = 1$.

- The q_4 parameter, introduced initially for water ice structures [P.-L. Chau, A. J. Hardwick, *Mol. Phys.* **1998**, 93, 511–518]:

$$q_4 = 1 - \frac{3}{8}\sum_{i>k}\left(\frac{1}{3} + \cos\alpha_{ijk}\right)^2$$

where the sum runs over the i-th and k-th ligand atoms surrounding the central atom j.

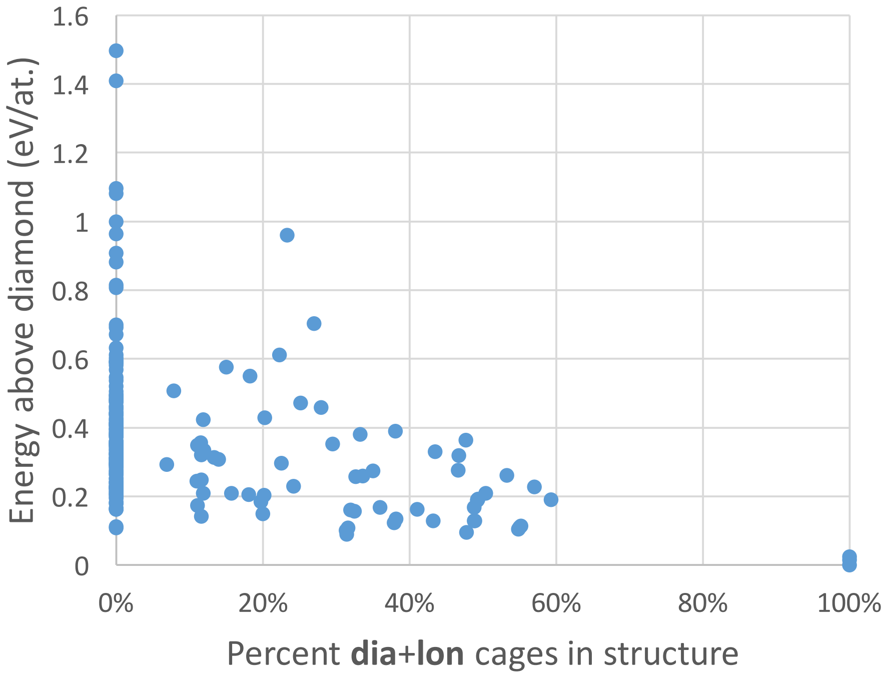

Figure S7: Contribution of dia and lon cages to the structures discussed, and the relation of this with energetic stability. We can take the tiling approach (see main text) a step further by evaluating the volume taken by each cage, and thereby quantify a percentage contribution of **dia** and **lon** cages to the total unit-cell volume. If this is large, it leads to lower energies as **dia** and **lon** cages "dilute" the other structural fragments. By contrast, carbon allotropes without *any* **dia** and **lon** contributions are found over a broad range of energies (far left).

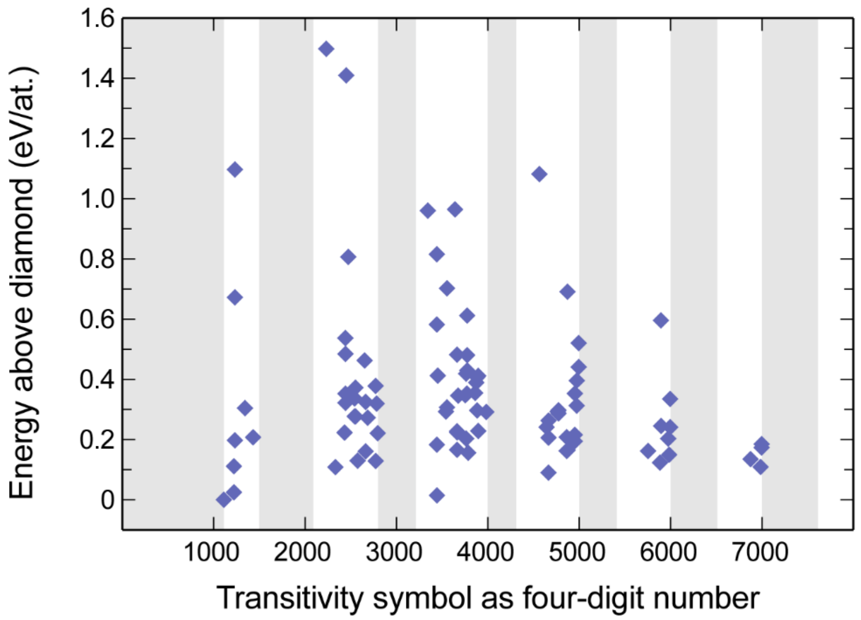

Figure S8: No direct correlation between structural simplicity and energetic stability. We here expand upon the question whether "simpler" carbon allotropes are generally more stable. A measure for structural simplicity can be obtained from the transitivity symbol [$N_n N_e N_r N_c$], giving the number of independent nodes, edges, rings, and cages (see above). The highest-order measure is thereby N_n, followed by N_e, and so on. Hence, reading the transitivity symbol as a four-digit number directly enables a qualitative ranking of the allotropes' simplicity (this is straightforward for all $N \leq 9$, to which we restrict our analysis here).

For example, **dia** is the simplest net (transitivity symbol [1111] → rank 1111), followed by **unj** (rank 1221), **lon** (rank 1222), and **crb**, **uni**, and **unc** (all rank 1232: note how different nets can have the same transitivity). These examples alone prove that no correlation exists between structural simplicity and energetic stability: dia is the most stable carbon allotrope with fourfold coordination, whereas **unc** (rank 1232) already lies more than 1 eV/at. above it; similarly, some more complex nets such as **cbn** (rank 4863) have very low energy (0.16 eV/at. above diamond). The plot above provides a more comprehensive perspective on this.[1]

[1] Furthermore, there are restrictions on the accessible transitivity symbols: First, for N_n nodes there must be at least (N_n – 1) edges, because these nodes need to be connected in the primitive cell. Furthermore, in all-fourfold coordinated structures, there is also a limit on the maximum number of edges given by $N_e \leq (3N_n + 1)$. Both restrictions are indicated in the plot by gray shading.

Overview of all structures found

Table S1. Structures found in our search that are known from SACADA. The leftmost column contains a running index (assigned in the order that structures were found; the index is therefore more or less arbitrary). For each structure, the table provides the number of independent nodes *N*, the topology, transitivity, and tiling symbols.

	Δ*E*	space group	*V* (A^3/at.)[a]	*N*	topol.	transi-tivity	tiling
18	**0.000**	$Fd\bar{3}m$	5.6903	1	**dia**	[1111]	[6^4]
143	**0.025**	$P6_3/mmc$	5.7043	1	**lon**	[1222]	[6^3]+[6^5]
115	**0.090**	*Cmcm*	5.8773	4	**znp**	[4664]	[5^2.6^2]+2[6^4]+[6^2.7^2]+[5^2.6^2.7^2]
17	**0.101**	*C*2/*m*	5.8622	6	4^6T7	[6(10)(10)5]	[5^2.6^2]+2[6^4]+[6^2.7^2]+[5^2.6^2.7^2]
45	**0.108**	$P4_2/ncm$	5.9532	2	**cdp**	[2332]	[5^2.7^2]+[5^2.6^2.7^2]
8	**0.109**	$Pmn2_1$	5.8659	6	4^6T15	[6985]	[6^3]+[5^2.6^2]+[6^2.7^2]+[6^5]+[5^2.6^2.7^2]
30	**0.111**	$P6_122$	6.2134	1	**unj**	[1221]	[5^2.8^2]
62	**0.128**	*C*2/*m*	5.8419	7	4^7T8	[7(12)(11)6]	[5^2.6^2]+3[6^4]+[6^2.7^2]+[5^2.6^2.7^2]
82	**0.129**	*Cmca*	5.8564	2	4^2T110	[2773]	2[6^3]+2[6^5]+[4^2.6^8]
144	**0.130**	*Cmmm*	5.8526	2	**sie**	[2575]	2[6^3]+2[6^2.8^2]+2[6^5]+[4^2.6^4]
66	**0.150**	*C*2/*m*	5.9000	5	4^5T14	[5984]	[5^2.6^2]+[6^4]+[6^2.7^2]+[5^2.6^2.7^2]
98	**0.156**	*Cmcm*	5.9065	3	4^3T87	[3785]	[6^3]+2[6^2.8^2]+[6^5]+[4^2.6^4]
97	**0.161**	*Imma*	5.9081	2	**byl**	[2663]	[6^3]+[6^5]+[4^2.6^8]
25	**0.162**	$I\bar{4}$	6.1043	5	4^5T15	[5753]	4[5^2.7^2]+[5^8]+[5^8.7^8]
3	**0.162**	*C*2/*m*	5.9624	4	**cbn**	[4863]	[5^2.6^2]+[6^2.7^2]+[5^2.6^2.7^2]
108	**0.165**	*P*2/*m*	5.9826	4	4^4T35	[4873]	[5^2.6^2]+[6^2.7^2]+[5^2.6^2.7^2]
1	**0.185**	*Amm*2	6.0282	6	4^6T6	[6996]	[6^3]+2[5^2.6^2]+[6^2.9^2]+[6^5]+[6^3.9^2]+[5^6.6^3]
9	**0.197**	*I*4/*mmm*	6.0111	1	**crb**	[1232]	[6^2.8^2]+[4^2.6^4]
109	**0.203**	*C*2/*m*	5.9537	3	4^3T85	[3763]	[5^2.6^2]+[6^2.7^2]+[5^2.6^2.7^2]
31	**0.203**	$P2_1/m$	5.9308	5	4^5T13	[5974]	[5^2.6^2]+[6^4]+[6^2.7^2]+[5^2.6^2.7^2]
202	**0.206**	$P222_1$	6.0336	4	4^4T75	[4664]	[5^2.6^2]+[6^4]+2[5^2.8^2]+[6^2.8^2]
73	**0.208**	*Cmca*	6.0174	1	**cag**	[1431]	[4^2.6^8]
2	**0.223**	*Cmcm*	6.1629	2	**bik**	[2432]	[5^2.6^2]+[5^2.6^2.8^2]
83	**0.230**	*C*2/*m*	6.0553	4	4^4T37	[4(12)(12)6]	4[6^3]+2[4.5^2.6^2]+2[4.6^3.7^2]+[5^4.6^4]+[6^6.7^4]
141	**0.304**	*I*4/*mcm*	6.3919	1	**tzs**	[1343]	2[6^3]+[4^2.6^2]+[6^4.8^2]
23	**0.307**	$C222_1$	5.7289	3	4^3T84	[3553]	2[6.7^2]+[6^2.7^2]+[5^2.6^4]
16	**0.337**	*C*2/*c*	5.8178	2	4^2T112	[2542]	[6^2.8^2]+[5^2.6^4]
155	**0.672**	$P6_122$	5.8205	1	**uni**	[1232]	[6^3]+[6.8^2]
114	**1.097**	$P4_122$	5.9077	1	**unc**	[1232]	[6^3]+[6.8^2]
4	**1.498**	*Cmmm*	6.1722	2	**mog**	[2232]	[4^2.8^2]+[6^4.8^2]

[a]We report here unit-cell volumes per atom; the density can be directly calculated using the conversion relation $\rho = (19.944\ \mathrm{g/cm^3})/V$, where the volume is in Å^3/at.

Table S2. As Table S1, but for networks that are known from other sources (see below).

	ΔE	space group	V (A^3/at)	N	topol.	transi-tivity	tiling	known from
153	**0.014**	$R\bar{3}m$	5.6984	3	**cfe**	[3443]	[6^3]+[6^4]+[6^5]	[a]
218	**0.166**	*Fmmm*	6.2213	3	4^3T141	[3663]	2[5^2.7^2]+2[5^2.6^2.7^2]+ [4^2.5^8.6^4]	[b]
206	**0.180**	$P\bar{1}$	6.1634	8	4^7T17	[8(18)(11)4]	2[5^2.7^2]+2[5^2.6^2.7^2]+ [4^2.5^16.7^4]	[b]
5	**0.183**	$I\bar{4}2d$	6.2577	3	4^3T143	[3442]	[5^2.8^2]	[b]
120	**0.195**	$P2_1/c$	5.9291	4	4^4T85	[4951]	[5^8.6^10]	[b]
59	**0.208**	*C*2/*c*	5.9000	4	4^4T86	[4863]	2[5^2.7^2]+[5^2.6^2.7^2]+ [5^2.6^4.7^2]	[b]
88	**0.229**	*Immm*	6.0473	3	4^3T144	[3895]	4[6^3]+2[4.5^2.6^2]+2[4.6^3.7^2]+ [5^4.6^4]+[6^6.7^4]	[b]
232	**0.272**	*Cmma*	6.1004	2	**atv**	[2685]	3[6^3]+[4^2.6^2]+[6^5]+[6^4.8^2]	[c]
80	**0.277**	$I4_1/a$	5.9509	2	4^2T193	[2543]	2[6^3]+[5^4.6^2]+[5^4.6^8]	[b]
158	**0.277**	*Imma*	6.1112	2	**mbc**-derived	[2552]	2[6^4]+[4^2.6^2.8^2]	[e]
227	**0.293**	*Pnma*	6.1095	3	4^3T91	[3542]	[6.7^2]+[4^2.6^3.7^2]	[d]
28	**0.322**	*Fmmm*	6.1310	2	**bcq**	[2442]	2[6^2.8^2]+[4^4.6^6]	[c]
51	**0.352**	*Pmma*	6.2636	2	**jbw**	[2442]	[6^4]+[4^2.6^2.8^2]	[c]
60	**0.372**	*C*2/*c*	6.4043	2	4^2T10	[2551]	[4.5^2.6.8^2]	[d]
26	**0.537**	*C*2/*c*	6.3493	2	**noq**	[2441]	[4^2.5^2.6^2.8^2]	[c]
138	**0.960**	*Cmmm*	6.0006	3	4^3T3	[3343]	[4^2.8^2]+2[6^4]+[6^4.8^2]	[d]
154	**1.410**	*C*2/*m*	6.3263	2	**nbo-x-d** derived	[2451]	[4.5^2.6^2.8^2]	[f]

[a] **cfe** (9-layered SiC polytype, consisting purely of dia and lon cages)
[b] known from zeolites (see references in the main text)
[c] known in RCSR (http://rcsr.net), but not yet described for carbon allotropes
[d] known from metal–organic frameworks (MOFs; in the TTD ToposPro database)§
[e]* full name: **mbc-**4,4-*C*2/*c*
[f]* full name: **nbo-x-d-**4,4-*C*2/*m*

§ See "Underlying nets in three-periodic coordination polymers: topology, taxonomy and prediction from a computer-aided analysis of the Cambridge Structural Database": E. V. Alexandrov, V. A. Blatov, A. V. Kochetkov, D. M. Proserpio, *CrystEngComm* **2011**, 13, 3947–3958.

* See "Topological relations between three-periodic nets. II. Binodal nets": V. A. Blatov, D. M. Proserpio, A*cta Crystallogr., Sect. A* **2009**, 65, 202–212.

Table S3. As Table S1, but for networks that are not known from SACADA or the other above-mentioned sources ("**new structures**"). These structures are labelled as **G*i*** in the manuscript, where ***i*** denotes the running index in the first column.

	Δ*E*	**space group**	*V* (A^3/at)	*N*	**transi-tivity**	**tiling**
225	**0.096**	*C*2/*m*	5.8370	8	[8(14)(12)7]	[5^2.6^2]+4[6^4]+[6^2.7^2]+[5^2.6^2.7^2]
12	**0.105**	*P*2$_1$/*m*	5.9091	7	[7(12)(10)6]	[5^2.6^2]+4[6^4]+[5^2.6^2.8^2]
14	**0.110**	*Cm*	5.9116	14	[(14)(21)(19)(12)]	2[6^3]+2[5^2.6^2]+4[6^4]+[6^2.8^2]+2[6^5]+[5^4.6^2.8^2]
94	**0.114**	*C*2/*m*	5.9099	7	[7(12)(11)6]	2[6^3]+[5^2.6^2]+2[6^5]+[5^2.6^2.8^2]
21	**0.123**	*P*2$_1$/*m*	5.9761	5	[5884]	[5^2.6^2]+2[6^4]+[5^2.6^2.8^2]
6	**0.135**	*Imm*2	5.9824	6	[6875]	2[6^3]+2[5^2.6^2]+[6^2.8^2]+2[6^5]+[5^4.6^2.8^2]
170	**0.142**	*Cm*	5.9167	16	[(16)(24)(21)(13)]	[6^3]+3[5^2.6^2]+2[6^4]+3[6^2.7^2]+[6^3.7^2]+[5^2.6^4]+2[5^2.6^2.7^2]
213	**0.162**	*P*2$_1$/*m*	5.8756	7	[7(11)(11)6]	[5^2.6^2]+3[6^4]+[6^2.7^2]+[5^2.6^2.7^2]
173	**0.167**	*P*$\bar{1}$	5.7875	8	[8(18)(14)5]	2[5^2.6^2]+6[6^4]+[5^4.6^14]
95	**0.168**	*Imm*2	5.8784	8	[8(11)(10)7]	[6^3]+[5^2.6^2]+[6^4]+[6^2.7^2]+[6^5]+[5^2.6^2.7^2]
241	**0.173**	*C*222$_1$	6.1019	6	[6996]	[5^2.6^2]+[6^4]+5[5^2.8^2]+[6^2.8^2]
128	**0.181**	*C*2/*m*	5.9622	5	[5(12)(10)4]	[5^2.6^2]+[6^2.7^2]+[5^2.6^2.7^2]
178	**0.188**	*P*2$_1$	5.7244	8	[8(16)(16)8]	2[6.7^2]+4[6^4]+[6^2.7^2]+[5^2.6^4]
79	**0.191**	*C*2	5.7283	8	[8(17)(17)8]	2[6.7^2]+4[6^4]+[6^2.7^2]+[5^2.6^4]
135	**0.191**	*C*2/*m*	5.9548	8	[8(13)(13)7]	[5^2.6^2]+5[6^4]+[5^2.6^2.8^2]
81	**0.192**	*P*1	5.7258	16	[(16)(32)(32)(16)]	[6^3]+2[6.7^2]+2[6^4]+[6^2.7^2]+[6^5]+[5^2.6^4]
165	**0.208**	*Cm*	5.9136	16	[(16)(24)(21)(13)]	[6^3]+3[5^2.6^2]+2[6^4]+3[6^2.7^2]+[6^3.7^2]+[5^2.6^4]+2[5^2.6^2.7^2]
92	**0.209**	*P*2$_1$	5.7223	8	[8(16)(16)8]	2[6^3]+2[6.7^2]+[6^2.7^2]+2[6^5]+[5^2.6^4]
40	**0.210**	*P*1	5.8170	6	[6(14)(10)3]	2[5^2.6^2]+2[6^4]+[5^4.6^14]
157	**0.212**	*P*1	6.1500	10	[(10)(20)(11)1]	[4^2.5^12.6^6.7^2]
230	**0.216**	*P*2$_1$/*c*	5.8900	4	[4951]	[5^8.6^10]
234	**0.221**	*Ibam*	5.9949	2	[2795]	4[6^3]+[4^2.6^2]+2[6^5]+[6^4.8^2]
22	**0.224**	*Imma*	6.0444	3	[3664]	[6^3]+[5^2.6^2]+[6^3.8^2]+[5^2.6^4]
151	**0.224**	*P*$\bar{1}$	5.9856	6	[6(14)92]	2[5^2.6^2]+[4^2.5^4.6^16]
171	**0.225**	*P*1	6.0506	16	[(16)(32)(26)(10)]	[5^2.6^2]+3[5^2.7^2]+2[7^4]+2[5^2.6.7^2]+[5^8]+[5^4.7^6]
237	**0.228**	*Pmma*	6.0196	3	[3663]	3[6^4]+[4^2.6^2.8^2]
169	**0.230**	*Cm*	5.9858	16	[(16)(24)(21)(13)]	[6^3]+3[5^2.6^2]+4[6^4]+[6^2.7^2]+[6^3.8^2]+[5^2.6^4]+[5^2.6^2.7^2]+[5^2.6^2.8^2]
210	**0.230**	*C*2	6.0005	8	[8(18)(12)2]	[5^2.6^2]+[4^2.5^2.6^12]

180	**0.237**	$P\bar{1}$	6.0487	8	[8(20)(13)5]	[5^2.7^2]+2[5.7^3]+[5^3.7^3]+[5^5.7^3]
78	**0.241**	$C222_1$	6.0067	5	[5995]	4[5^2.7^2]+[6^2.7^2]+2[5^2.8^2]+[6^2.8^2]
105	**0.241**	*Pmmn*	6.1897	4	[4643]	2[5^2.6^2]+[6^2.8^2]+[5^4.6^2.8^2]
125	**0.242**	*C*2	5.9495	9	[9(17)(14)6]	5[5^2.7^2]+2[6^2.7^2]+2[5.6^2.7^3]+[5^8.6^4]
93	**0.245**	*Cmmm*	5.9992	5	[5896]	[5^2.6^2]+2[6^4]+2[6^2.8^2]+2[6^3.8^2]+[5^8.6^4]
205	**0.248**	$P2_1/m$	6.0050	8	[8(13)(11)6]	2[6.7^2]+[5^2.6^2]+[6^4]+[4^2.6^3.7^2]+[5^2.6^3.7^2]
89	**0.252**	$P\bar{1}$	5.8822	8	[8(18)(12)3]	2[5^2.6^2]+2[6^4]+[4^2.5^4.6^20]
204	**0.253**	*P*1	6.0484	16	[(16)(32)(25)9]	[5^2.7^2]+2[5.7^3]+5[5^3.7^3]+[5^5.7^3]
63	**0.257**	$P2_1$	5.7414	6	[6(12)(12)6]	2[6.7^2]+2[6^4]+[6^2.7^2]+[5^2.6^4]
7	**0.259**	*C*2	5.7251	6	[6(13)(13)6]	[6^3]+2[6.7^2]+[6^2.7^2]+[6^5]+[5^2.6^4]
86	**0.262**	$C222_1$	5.7493	5	[5(10)(10)5]	2[6.7^2]+4[6^4]+[6^2.7^2]+[5^2.6^4]
27	**0.263**	$I2_12_12_1$	5.9200	4	[4664]	[5^2.6^2]+2[5^2.7^2]+[6^2.7^2]+[7^4]
163	**0.264**	$P\bar{1}$	5.9656	8	[8(18)(13)6]	4[6.7^2]+2[5^2.6^2]+[4^2.6^4]+[5^2.6^6.7^2]+[4^2.5^2.6^8.7^2]
177	**0.269**	*P*1	6.0517	16	[(16)(32)(23)7]	[5^2.7^2]+2[5.7^3]+2[5^3.7^3]+[5^5.7^3]+[5^9.6^2.7^3]
112	**0.274**	$P\bar{1}$	5.7963	5	[5(12)(10)5]	[6^3]+[6^2.8^2]+[6^5]+[5^2.6^4]
162	**0.283**	*P*1	5.9293	16	[(16)(32)(26)(10)]	2[5^2.6^2]+3[5^2.7^2]+2[6^2.7^2]+[7^4]+[5^2.6^2.7^2]+[5^6.6^6.7^2]
24	**0.287**	$C222_1$	5.8726	4	[4774]	2[5^2.6^2]+[5^2.7^2]+2[6^2.7^2]+[7^4]
228	**0.291**	$P2_1/m$	6.2445	3	[3983]	[6^3]+[4.5^2.6^2]+[4.5^2.6^5.8^2]
54	**0.293**	*P*2	5.8764	8	[(16)(28)(28)(16)]	4[5^2.6^2]+[6^4]+2[5^2.7^2]+6[6^2.7^2]+[7^4]
229	**0.293**	*Cmcm*	6.2145	4	[4764]	[5^2.6^2]+[6^4]+[4^2.6^2.8^2]+[5^2.6^2.8^2]
149	**0.296**	$P\bar{1}$	6.2062	6	[6(14)92]	2[5^2.6^2]+[4^2.5^12.6^4.7^4]
58	**0.297**	*Ima*2	6.2459	3	[3883]	[6^3]+[4.5^2.6^2]+[4.5^2.6^5.8^2]
107	**0.297**	*C*2/*m*	6.1041	4	[4773]	[5^2.6^2]+[6^4]+[5^2.6^2.8^2]
90	**0.298**	*C*2/*c*	5.7965	5	[5(10)95]	[6^3]+[6^4]+[6^2.8^2]+2[6^5]+[5^2.6^4]
91	**0.301**	*C*2	6.0677	8	[8(17)(13)6]	2[4.6^2]+2[6^3]+2[5^2.6^2]+[6^5]+[4^2.5^8.6^9.7^4]
11	**0.308**	$P\bar{1}$	5.8165	7	[7(16)(12)3]	[5^2.6^2]+[6^4]+[5^2.6^10]
159	**0.310**	*C*2/*m*	5.8251	6	[6(12)(11)5]	[6^3]+2[6.7^2]+[6^5]+2[5.6^3.7]+[5^2.6^2.7^2]
176	**0.312**	*Cc*	5.9577	8	[8(16)(10)2]	[6^3]+[5^8.6^9]
208	**0.313**	$P2_1/c$	5.9388	4	[4972]	[5^2.6^2]+[5^2.6^4.7^2]
156	**0.314**	*P*1	5.8986	14	[(14)(28)(17)3]	2[5^2.6^2]+[5^8.6^18]
69	**0.314**	*C*2	5.9335	8	[8(15)(11)5]	3[5^2.6^2]+2[6^4]+[5^2.6^6]+[5^4.6^8.7^2]
76	**0.319**	*Cmcm*	6.1578	2	[2783]	2[6^3]+2[6^5]+[4^4.6^4.8^2]
179	**0.321**	*Amm*2	6.1475	5	[5(12)(12)6]	3[6^3]+[4.5^2.6^2]+2[4.6^3.7^2]+[4^3.5^2.6^2]+[5^4.6^7.7^4]
134	**0.322**	$P2_1$	5.9492	8	[8(16)(11)3]	[5^2.6^2]+[6^4]+[5^6.6^6.7^2]

85	**0.324**	$P\bar{1}$	5.9834	8	[8(18)(15)6]	4[6.7^2]+2[5^2.6^2]+2[4^2.6^4]+[5^2.6^2.7^2]+2[5.6^4.7^3]
99	**0.325**	*Cmcm*	6.2557	2	[2663]	[6^3]+[4.5^2.6^2]+[4.5^2.6^5.8^2]
182	**0.325**	*Cc*	5.9931	6	[6(12)71]	[5^8.6^4.7^2]
68	**0.330**	*C*2	5.7730	7	[7(15)(15)7]	2[6.7^2]+3[6^4]+[6^2.7^2]+[5^2.6^4]
117	**0.332**	$P2_1/c$	5.7746	4	[4(10)51]	[5^2.6^8]
123	**0.335**	$C222_1$	5.9259	5	[5995]	2[5^2.6^2]+[5^2.7^2]+[6^4]+4[6^2.7^2]
121	**0.339**	*P*1	5.9560	16	[(16)(32)(29)(13)]	4[6.7^2]+[5^2.6^2]+2[5^2.7^2]+[6^2.7^2]+2[5.6^2.7.8]+[4.5.6^3.7]+[4.6^3.7^2]+[5^3.6^4.7]
100	**0.346**	*Cmmm*	6.1459	3	[3675]	2[5^2.6^2]+2[6^2.8^2]+2[6^3.8^2]+[4^2.5^4.6^2]
146	**0.347**	*C*2/*m*	6.4115	3	[3753]	[5.7.8^2]+[4^2.5^4]+[4^2.7^4]
136	**0.349**	*P*1	5.9153	16	[(16)(32)(21)5]	2[5^2.6^2]+2[6^4]+[5^8.6^18]
61	**0.349**	$P\bar{1}$	5.9737	7	[7(16)(12)5]	[5^2.6^2]+[5^2.7^2]+[5^2.6^2.7^2]+[5^2.6^4.7^2]
187	**0.353**	*C*2/*c*	6.3235	3	[3772]	[5^2.8^2]+[4.5^2.6.8^2]
207	**0.353**	$P2_1/c$	6.4233	4	[4952]	[4^2.5^4]+[4^2.5^4.7^8]
167	**0.354**	*P*2/*c*	5.9802	5	[5(10)(10)5]	2[6.7^2]+[5^2.6^2]+2[5^2.7^2]+2[6^2.7^2]+[5^2.6^2.7^2]
219	**0.355**	*Ama*2	6.4637	3	[3861]	[4^4.5^4.6^4.8^2]
119	**0.356**	$P2_1$	5.9174	8	[8(16)(11)3]	[5^2.6^2]+[6^4]+[5^4.6^10]
77	**0.358**	*C*2	6.1669	9	[9(17)(11)4]	3[5^2.7^2]+[5^2.6^2.7^2]+[4^2.5^16.6^2.7^4]
118	**0.359**	*P*1	5.9947	16	[(16)(32)(23)7]	2[6.7^2]+2[5^2.7^2]+[4.6^3.7^2]+[5^6.6^6]+[4.5^4.6^7.7^2]
132	**0.361**	$P\bar{1}$	6.1711	8	[8(18)(11)3]	2[4.5^2.6.7^2]+[5^2.6^6.7^2]+[4^2.5^10.6^4.7^2]
137	**0.363**	*C*2/*m*	5.8294	4	[4(10)94]	[6^4]+[6^2.8^2]+[5^2.6^4]
172	**0.375**	*P*1	6.1507	16	[(16)(32)(21)5]	[4.5^2.6.7^2]+[4.5.6.7^3]+[4^2.5^4.7^4]+[4.5^3.6^3.7^3]+[4.5^2.6^5.7^2]
164	**0.378**	*Imma*	6.3241	2	[2772]	[4^4.6^4.8^2]+[4^2.6^8]
174	**0.379**	*Cc*	5.9126	8	[8(16)(13)5]	[5^2.6^2]+2[6^2.7^2]+[5^2.6.7^2]+[5^4.6^3.7^2]
87	**0.380**	$P2_1$	5.8784	8	[8(16)(11)3]	[6^3]+[5^2.6^3]+[5^4.6^10]
197	**0.381**	*P*2	5.7935	6	[6(14)(14)6]	[6^3]+2[6.7^2]+[6^2.7^2]+[6^5]+[5^2.6^4]
231	**0.383**	*Pm*	6.3498	6	[6(15)(17)8]	[6^3]+2[6.8^2]+[4.5^2.6^2]+[6.8^4]+[4^3.5^2.6^2]+[4^2.6^3.8^2]+[5^4.6.8^2]
220	**0.386**	*Cm*	6.3348	6	[6(14)(14)6]	[6^3]+2[6.8^2]+[4.5^2.6^2]+2[5^2.6.8^3]+[4^3.5^2.6^2]+[4^2.6^3.8^2]
129	**0.388**	*P*1	5.9489	16	[(16)(32)(25)9]	[6^3]+3[6.7^2]+[5^2.6^2]+[5^2.7^2]+[5.6.7^3]+[5^5.6^2.7^3]+[5^8.6^5.7^2]
39	**0.389**	*C*2/*c*	5.8324	3	[3873]	[6^4]+[6^2.8^2]+[5^2.6^4]
104	**0.396**	*C*2/*m*	6.1682	4	[4973]	4[6^3]+2[4.5^2.6^2]+[5^4.6^12]
124	**0.396**	$P\bar{1}$	5.9309	8	[8(19)(12)3]	[6^3]+[5^2.6^2]+[5^4.6^11]
64	**0.397**	$P2_1$	6.1860	6	[6(12)71]	[4^2.5^6.6^4.7^2]
161	**0.405**	*P*1	5.9759	16	[(16)(32)(19)3]	[5^2.6^2]+[4.5^2.6^3]+[4.5^8.6^19]
217	**0.407**	*C*2/*c*	6.0872	3	[3883]	2[5^2.6^2]+2[5.7.8^2]+[5^2.6^2.7^2]
131	**0.409**	*C*2	5.9854	9	[9(16)(16)9]	2[5^2.6^2]+3[6^4]+[5^2.8^2]+2[6^2.8^2]

175	**0.412**	*Fmmm*	6.2208	3	[3895]	[6^2.8^2]+[4.5^2.6^2]+[4.7^2.8^2]+[4^2.7^4]+ [5^2.6^2.7^2]
96	**0.412**	$I\bar{4}2d$	5.9401	3	[3452]	2[6.7^2]+[4.5^2.6^2.7^2]
238	**0.412**	*C*2/*m*	6.0320	3	[39(11)5]	2[6^3]+2[6.8^2]+4[6^2.8^2]+3[4^2.6^4]
35	**0.413**	$P\bar{1}$	6.2002	5	[5(12)72]	[4^2.7^4]+[4^2.5^8.6^4.7^4]
184	**0.419**	*C*2/*c*	6.0874	3	[3763]	[6^3]+[5^2.6^2.7^2]+[4^2.5^2.6^3.7^2]
166	**0.423**	*P*1	5.9594	16	[(16)(32) (25)9]	3[5^2.6^2]+[5^2.7^2]+2[6^4]+2[6^2.7^2]+ [5^6.6^10.7^2]
126	**0.423**	$P\bar{1}$	5.8552	8	[8(17) (13)3]	2[6^3]+2[4.5^2.6^3]+[5^4.6^20]
130	**0.428**	*C*2	6.1672	8	[8(17) (13)5]	2[4.7^2]+2[6.7^2]+[5^2.6^2.7^2]+ 2[4^2.5^4.6^2.7^2]+[5^2.6^6.7^2]
194	**0.429**	$C222_1$	5.7772	3	[3774]	3[6^3]+[6^4]+[6^7]
13	**0.436**	*P*1	6.1892	8	[8(16)91]	[4^2.5^8.6^6.7^2]
152	**0.440**	$P2_1$	6.3090	6	[6(12) (12)6]	2[4.6^2]+[6^3]+[4.8^2]+[6.8^2]+[4.6^6.8^2]
72	**0.441**	*C*2	5.7955	4	[4994]	2[6.7^2]+[6^2.7^2]+[5^2.6^4]
52	**0.442**	$P\bar{1}$	6.2428	6	[6(13) (10)1]	[4^3.5^4.6^5.7^2]
209	**0.443**	*C*2/*c*	5.8990	4	[4(10)85]	[6^3]+2[6.7^2]+2[5^2.6^2]+[5^2.6.7^2]+[5^2.7^6]
20	**0.454**	$P\bar{1}$	5.7714	4	[4(11)94]	2[6.7^2]+[6^2.7^2]+[5^2.6^4]
186	**0.458**	*C*2	6.0511	7	[7(14) (13)5]	4[6.8^2]+2[5.7.8^2]+[5^6]+2[5^4.6^2.7^2]
216	**0.460**	*P*1	5.8703	14	[(14)(28) (29)(15)]	2[6^3]+6[6.7^2]+3[6^2.7^2]+2[6^5]+[4^2.6^4]+ [5^2.6^4]
147	**0.463**	*C*2/*c*	5.9438	2	[2652]	[6^2.8^2]+[4^2.6^6]
133	**0.472**	*C*2	5.9924	9	[9(16) (11)4]	2[6^4]+[4^2.5^2.6^10]
122	**0.475**	$P\bar{1}$	5.9467	8	[8(19) (18)9]	[4.6^2]+[4.7^2]+[6^3]+5[6.7^2]+[5^2.6^6.7^2]
160	**0.476**	*P*1	6.1592	16	[(16)(32) (19)3]	[6.7^2]+[4.5^2.6^3]+[4^5.5^10.6^12.7^2]
145	**0.480**	*Cmm*2	6.7030	3	[3774]	2[6.8^2]+[4^2.8^2]+[4^4.6^2]+2[4.6^2.8^3]
190	**0.482**	*Cmma*	5.9839	3	[3663]	2[6^2.8^2]+[4^2.5^2.6^4]
116	**0.484**	*Fddd*	5.9614	2	[2442]	[6^3]+[5^2.6^2.7^2]
214	**0.491**	*P*1	6.1535	14	[(14)(28) (18)4]	[5^2.6^2]+[4^2.5^2.6^4.7^2]+[4^2.5^4.7^4]+ [5^4.6^6.7^2]
193	**0.494**	*C*2	5.9482	8	[8(15) (15)8]	3[5^2.6^2]+6[5^2.7^2]+[6^2.7^2]+4[7^4]
212	**0.496**	$P2_1$	6.1959	7	[7(14) (12)5]	[4.6^2]+[4.7^2]+2[6.7^2]+[4^2.5^2.6^4.7^4]
215	**0.505**	$P\bar{1}$	6.0080	7	[7(15) (15)6]	8[6.8^2]+2[4^3.6^4]+[4^2.6^10]
183	**0.507**	*C*2	6.0350	7	[7(12)72]	[6^4]+[4^4.5^4.6^16]
84	**0.520**	*Fdd*2	5.8330	4	[4994]	2[6.7^2]+[6^2.7^2]+[5^2.6^4]
222	**0.547**	$P\bar{1}$	6.2863	5	[5(12)82]	2[4.5^2.6.7^2]+[4^2.5^4.6^4.7^4]
198	**0.550**	*C*2	6.0720	6	[6(14) (12)4]	2[6^4]+2[6^2.8^2]+[5^2.6^4]+[4^4.6^4.7^2]
185	**0.570**	*P*1	6.0819	12	[(12)(24) (15)3]	2[5^2.6^2]+[4^2.5^6.6^12.7^2]
142	**0.577**	$P\bar{1}$	6.0253	6	[6(13) (13)4]	2[5^2.6^2]+[6^4]+[5^2.6^4.8^2]
47	**0.582**	*I*222	6.4109	3	[3442]	[6^2.10^2]+[4^4.5^8.10^2]
150	**0.589**	$P2_1$	6.0940	6	[6(12)71]	[4^2.5^4.6^6.7^2]

127	**0.591**	$P\bar{1}$	6.1257	8	[8(17)(13)5]	2[4.7^2]+2[6.7^2]+[4^2.5^4]+[5^2.6^2.7^2]+[4^2.5^6.6^8.7^6]
196	**0.591**	*C*2	6.1686	6	[6(14)(10)5]	2[4.7^2]+2[6^3]+[4^2.5^4]+[5^4.6^10.7^4]
103	**0.596**	*C*2/*m*	6.3024	5	[5894]	[4^2.8^2]+2[6^2.8^2]+[6.8^3]+[4^3.6^3]
244	**0.603**	$P\bar{1}$	6.1904	6	[6(13)(10)3]	2[4.6^2]+[6^2.7^4]+[4^6.5^4.6^6.7^4]
195	**0.612**	*C*2	6.0601	7	[7(12)(13)7]	2[8^3]+4[5^2.6^2]+3[6^4]+2[5^2.8^2]+2[6^2.8^2]
189	**0.612**	*C*2/*c*	6.3765	3	[3774]	2[4.6^2]+2[6.8^2]+[7^2.8^2]+[4^2.7^4]
191	**0.632**	*C*2	6.1778	6	[6(12)(10)5]	2[4.6^2]+2[6^3]+[4^2.5^2.6^4.7^2]+[5^2.6^4.7^4]
181	**0.691**	*P*2/*c*	6.2262	4	[4872]	[5.7.8^2]+[4^4.5^2.6^4.7^2]
221	**0.700**	*Cc*	6.1292	6	[6(12)(11)5]	[4.8^2]+2[6.7^2]+[6.8^2]+[4^3.5^2.6.7^2.8^2]
201	**0.702**	*C*222	5.6843	3	[3553]	[6^4]+[6^2.8^2]+[5^4.6^4.8^2]
102	**0.807**	*Cccm*	6.2126	2	[2473]	2[6.8^2]+[6^2.8^2]+2[4.5^2.8^2]
110	**0.815**	*Fmmm*	6.0992	3	[3442]	2[6^2.8^2]+[4^4.6^8]
245	**0.881**	$P\bar{1}$	6.0370	6	[6(13)(13)4]	[4.8^2]+2[6.8^2]+[4.5^4.6^4.8^2]
188	**0.909**	*P*1	6.2364	12	[(12)(24)(23)(11)]	2[4.9^2]+2[6.8^2]+[8^3]+[4^2.5.7.8]+[4.5.6^2.7]+[6^2.7^2.8]+[5.6.8^2.9]+[5.8^3.9]+[4^3.5^2.6]
113	**0.964**	*C*2	6.1271	3	[3641]	[4^4.5^2.7^4.9^2]
223	**1.000**	*Cc*	6.1079	5	[5(10)72]	[4^3.5.7.9^2]+[4.5.7^3.9^2]
199	**1.082**	*Cmmm*	6.1993	4	[4564]	2[4^2.8^2]+[5^4.8^2]+2[5^2.6^2.7^2]+[7^4.8^2]
